# The Good Reviewer's Guide to the Publishing Galaxy

**DOI:** 10.1523/ENEURO.0362-19.2019

**Published:** 2019-09-18

**Authors:** Christophe Bernard

**Affiliations:** Editor-in-Chief

After having spent months/years doing experiments and analyses, we are finally ready to tell our story to our fellow scientists. However, before knowledge is transmitted to others, we must pass under the yoke of the review process. In many instances, it is painful, stressful, and even, sometimes, humiliating. The “best” review I ever received was from one of the two *you-know-who* journals. Verbatim, the full review was “It is incredible if it is true.” I cherish it as a souvenir and use it as a perfect example of what must *not* be done. Quality in peer review is this year’s topic for Peer Review Week.

What is a good-quality review? The answer is surprisingly easy: a good review is a helpful and useful one. When early career scientists come to see me when they have a paper to review, sometimes for the first time, I tell them that the only thing they have to do is to check whether the interpretations/conclusions are supported by the presented data and analyses. If there are some issues, they must try to help the authors provide a better case without asking them to do unnecessary experiments.

Our review process at *eNeuro* is based on these basic principles. The reviewing editors pay great attention to what is transmitted to authors: reviews must be factual, not emotional, and should include improvement suggestions (if necessary). If the reviewers agree that more experiments are needed and that experiments will require more than two months’ work, the paper is automatically rejected (with the possibility to resubmit). This procedure allows researchers to really ponder which additional experiments are truly necessary. Finally, reviewers and the reviewing editor must reach a consensus on what comments will be transmitted to authors. Therefore, the authors receive a one-voice factual report. This provides a clear directive toward the path to publication and eliminates the need for authors to try to interpret the priorities of separate reviewers. Sometimes, generating one consensus review requires several exchanges and discussion between the reviewers and the reviewing editor, dialogue is the key to success. We know that the system works as, since the launch of *eNeuro* in 2014, I can count on two hands the number of appeals I have received. Even if one may be unhappy after rejection, the decision is accepted because the facts and reasons are provided. I am 100% convinced that this type of reviewing (pioneered by *eLife*) is today’s best solution to the concerns raised by traditional peer review. It is easy to implement, but it takes more time per manuscript. For obvious reasons, it works best if the reviewing editor is an active scientist. We also know that *eNeuro*’s system works based on the positive comments we receive from authors (included those with rejected papers) and reviewers regarding the quality of our peer review process. Before hopefully becoming the norm, mentalities must change.

The best way forward is to teach the young generations the fundamentals of a good-quality review. Unfortunately, there are few teaching courses provided by research institutions on how to review a paper. The Society for Neuroscience offers a mentorship program (https://www.jneurosci.org/content/sfn-reviewer-mentor-program) to train graduate students, postdocs, or established researchers to write good and helpful reviews; trainees are then invited to become reviewers at *eNeuro*. When you are a reviewing editor, you may think that you are taking a risk when selecting a non-seasoned reviewer. But so far, reviewing editors who have used trainees from the program have been enthusiastic regarding the quality of their reviews. I believe that this will induce a virtuous circle. The happier authors become with the review process, the better their own reviews will be, making even more authors happy, etc. I am not overly optimistic; it is working at *eNeuro*. We have the opportunity to shape the future of the publication field. Let us seize it.

And for those who have read the Guide, you know that the final answer is to be found on page 42.

